# Single-nucleus transcriptome sequencing reveals hepatic cell atlas in pigs

**DOI:** 10.1186/s12864-023-09765-9

**Published:** 2023-12-12

**Authors:** Jun-Hong Zhu, Xuan-Cheng Guan, Lan-Lan Yi, Hong Xu, Qiu-Yan Li, Wen-Jie Cheng, Yu-Xiao Xie, Wei-Zhen Li, Hong-Ye Zhao, Hong-Jiang Wei, Su-Mei Zhao

**Affiliations:** 1 Yunnan Key Laboratory of Animal Nutrition and Feed Science, Yunnan Agricultural University, Kunming, 650201 China; 2 Yunnan Province Key Laboratory for Porcine Gene Editing and Xenotransplantation, Yunnan Agricultural University, Kunming, 650201 China; 3 School of Public Finance and Economics, Yunnan University of Finance and Economics, Kunming, 650221 China; 4 College of Biology and Agriculture, Zunyi Normal University, Zunyi, 563006 China; 5 College of Veterinary Medicine, Yunnan Agricultural University, Kunming, 650201 China

**Keywords:** Hepatic tissue, Single-nucleus RNA sequencing, Cell atlas, Dahe pigs, Dahe black pigs

## Abstract

**Background:**

As the largest substantive organ of animals, the liver plays an essential role in the physiological processes of digestive metabolism and immune defense. However, the cellular composition of the pig liver remains poorly understood. This investigation used single-nucleus RNA sequencing technology to identify cell types from liver tissues of pigs, providing a theoretical basis for further investigating liver cell types in pigs.

**Results:**

The analysis revealed 13 cells clusters which were further identified 7 cell types including endothelial cells, T cells, hepatocytes, Kupffer cells, stellate cells, B cells, and cholangiocytes. The dominant cell types were endothelial cells, T cells and hepatocytes in the liver tissue of Dahe pigs and Dahe black pigs, which accounts for about 85.76% and 82.74%, respectively. The number of endothelial cells was higher in the liver tissue of Dahe pigs compared to Dahe black pigs, while the opposite tendency was observed for T cells. Moreover, functional enrichment analysis demonstrated that the differentially expressed genes in pig hepatic endothelial cells were significantly enriched in the protein processing in endoplasmic reticulum, MAPK signaling pathway, and FoxO signaling pathway. Functional enrichment analysis demonstrated that the differentially expressed genes in pig hepatic T cells were significantly enriched in the thyroid hormone signaling pathway, B cell receptor signaling pathway, and focal adhesion. Functional enrichment analysis demonstrated that the differentially expressed genes in pig hepatic hepatocytes were significantly enriched in the metabolic pathways.

**Conclusions:**

In summary, this study provides a comprehensive cell atlas of porcine hepatic tissue. The number, gene expression level and functional characteristics of each cell type in pig liver tissue varied between breeds.

**Supplementary Information:**

The online version contains supplementary material available at 10.1186/s12864-023-09765-9.

## Background

Pigs are one of the most farmed livestock in the world. The meat of pigs is widely eaten by people across the world [1]. At the same time, the physiological structure of pigs is similar to that of humans, so it is an ideal animal model [2]. The liver is the largest digestive gland in the animal body and plays an important role in the physiological processes of digestion, metabolism, and immune defense [3–5]. Furthermore, the liver is a visceral organ that is capable of remarkable natural regeneration after tissue loss and retain basic metabolic functions [6–8]. However, the liver cellular landscape has barely been explored at single-cell resolution, which limits our molecular understanding of pig liver structure and function.

Traditional transcriptomics detects the average value of all cells in a sample, and it is difficult to give a clear answer at the cellular scale [9]. Single-nucleus RNA sequencing (snRNA-seq), enables robust and unbiased exploration of individual cell states and types, yielding new insights into tissue biology [10–12]. Therefore, snRNA-seq is a promising approach to investigate the transcriptome of individual cells in tissue [13]. Recent studies have shown that snRNA-seq has emerged as a complementary approach to investigate complex tissues at single-cell level in mouse and human samples [14–20]. However, the application of snRNA-seq in pigs is relatively limited. Recent snRNA-seq analysis were focused on the lung, cerebral cortex, hypothalamus, and peripheral blood in pigs [21–23]. There is limited information on the liver atlas of pigs.

Therefore, this study used snRNA-seq to identify cell types, explore the biological function of pig liver deeply, and describe the transcriptome characteristics of cells comprehensively. The findings revealed the transcriptional landscape of the pig liver and the effect of pig breeds on liver cell types and functions, providing a theoretical basis for future in-depth research on pig liver function.

## Materials and methods

### Animal feed and liver tissue collection

In the Dahe Black Pig Research Institute of Fuyuan County, six male Dahe pigs and six male Dahe black pigs of the same batch with similar body weight were randomly selected. Pigs of the same breed come from the same maternal parent and parity. Each pig was raised in a single pen and all pigs were fed the same basic diet without any antibiotics. When pigs reached 194 days of age, the right lobe of the liver was collected from every animal, a part was fixed with 4% paraformaldehyde fixative solution for histological verification, and the rest was frozen in liquid nitrogen for the preparation of single nuclear suspension and RNA extraction.

### Liver morphology and cell number

Liver samples were removed from the 4% paraformaldehyde fixative solution and embedded in paraffin. Each liver was sliced to a thickness of 4 mm. From each block, two consecutive histological sections randomly positioned within the block and mounted on adhesion microscope slides. Sections were stained with hematoxylin and eosin (Beijing Solarbio Science&Technology Co., Ltd., Beijing, China) for analysis. Periodic acid-Schiff staining (PAS) (Shanghai Yuanye Biotechnology Co., Ltd., Shanghai, China) was used to highlight the contours of the individual cell and to count the endothelial cell, hepatocyte, and lymphocyte.

### Cell suspension preparation

The liver tissue samples for snRNA-seq were selected from one male Dahe pigs and one male Dahe black pigs with body weights closest to the average body weight. Samples were subjected to nuclear isolation, sequencing, and library preparation following the 10X Genomics protocol. Approximately 500 mg of hepatic tissue was dissociated into a singular nuclear suspension via tissue homogenization in a chilled lysis buffer (0.25 M sucrose, 5 mM CaCl_2_, 3 mM MgAc_2_, 10 mM Tris–HCl pH 8.0, 1 mM DTT, 0.1 mM EDTA, 1 × Protease Inhibitor and 1U/µL RiboLock RNase Inhibitor (Thermo Scientifc, cat no. O0381) with pestle strokes. The resulting homogenate was subsequently filtered through a 70 µm cell strainer, yielding a nuclear fraction collected in a 50 ml centrifuge tube, with a 1 ml volume. This nuclear fraction was mixed with an equal volume of 50% iodixanol solution (0.16 M sucrose, 10 mM NaCl_2_, 3 mM MgCl_2_, 10 mM Tris–HCl pH 7.4, 1 U/µL RiboLock RNase Inhibitor, 1 mM DTT and 0.1 mM PMSF Protease Inhibito (Thermo Scientifc, cat no. 36978), yielding a final concentration of 25%, and supplemented with 1 mL of 33% iodixanol solution at the tube's base and 30% iodixanol solution at the top. The solution underwent inversion mixing 10 times before centrifugation at 500 × g for 8 min at 4℃, subsequent to myelin layer removal from the gradient’s apex. Nuclei were harvested from the 30% iodixanol interface, resuspended in nuclear wash and resuspension buffers, and centrifuge (0.04% bovine serum albumin, 0.2 U/µL RiboLock RNase inhibitor, 500 mM mannitol and 0.1 mM PMSF protease inhibitor in PBS) at 500 × g and 4℃ for 5 min. Filtration through a 40 µm cell filter eliminated cell debris and sizable aggregates. The nuclei’s total count, concentration, and integrity ratio were ascertained via hemocytometer-assisted microscopic examination of trypan-stained samples. Ultimately, the nuclear concentration was regulated to 700–1,200 nuclei/µL, with nuclei inspected using the 10X Chromium platform.

### snRNA-seq library preparation and sequencing

Cellular suspensions were loaded on a 10X Genomics GemCode Single-cell instrument that generates single-cell Gel Bead-In-EMlusion (GEMs). Using Chromium Next GEM Single Cell 3'Reagent Kit v3.1 for Library Generation and Sequencing of cDNA. Upon dissolution of the Gel Bead in a GEM, primers containing (i) an Illumina® R1 sequence (read 1 sequencing primer), (ii) a 16 nt 10 × Barcode, (iii) a 10 nt Unique Molecular Identifier (UMI), and (iv) a poly-dT primer sequence were released and mixed with cell lysate and Master Mix [24].

Silane magnetic beads were used to remove leftover biochemical reagents and primers from the post GEM reaction mixture. Full-length, barcoded cDNAs were then amplified by PCR to generate sufficient mass for library construction. R1 (read 1 primer sequence) were added to the molecules during GEM incubation. P5, P7, a sample index, and R2 (read 2 primer sequence) were added during library construction via End Repair, A-tailing, Adaptor Ligation, and PCR. The final libraries contained the P5 and P7 primers used in Illumina bridge amplification. A Single Cell 5’ Library comprised standard Illumina paired-end constructs which begin and end with P5 and P7. The Single Cell 5′ 16 bp 10X Barcode and 10 bp UMI were encoded in Read 1, while Read 2 was used to sequence the cDNA fragment. Sample index sequences were incorporated as the i7 index read. Read 1 and Read 2 were standard Illumina® sequencing primer sites used in paired-end sequencing [25].

### snRNA-seq data processing and quality control

FASTQ files were processed with 10X Genomics Cell Ranger (v3.1.0) using the default parameters and aligned to the Ensembl_release104 Sscrofa 11.1 (NCBI Accession AEMK00000000.2) reference genomes. The gene expression matrix was processed and analyzed by Seurat package (v3.2.1). To filter out low-quality cells, the investigation removed reads from cells in which less than 240 and over 3,600 genes were detected, cells with more than 16,000 UMIs, and cells with more than 10% share of mitochondrial gene expression.

### Cell clustering by Seurat

Using the R package Seurat [26], multiple criteria were employed to filter cells, eliminating multi-cellular entities and cells with suboptimal states, while retaining high-quality cells. The gene expression levels were normalized using a log transformation method, with “mitochondrial genes” and “cell cycle scoring” as regression variables. The resulting high-quality cell data was subjected to data integration and batch effect correction using Harmony [27]. Soft k-means clustering was applied to the dimension-reduced data.

To mitigate noise interference from individual gene expression levels in single-cell transcriptomics data, the vst algorithm was applied for high-variable gene selection. Principal Component Analysis (PCA) was performed on the integrated data to reduce the dimensionality to 50 principal components for capturing the main information. The parameter k.param was set to 20, and the Annoy algorithm with Euclidean distance metric was employed to calculate cell-to-cell distances. The Louvain [28] algorithm was used for clustering the dimension-reduced data with a resolution parameter set to 50, partitioning all cells in the single-cell transcriptome into distinct cell subpopulations, facilitating subsequent analyses.

### Differentially expressed genes (DEGs)

Expression value of each gene in given cluster were compared against the rest of cells using Wilcoxon rank sum test [29]. Significant up-regulated and down-regulated genes were identified using a number of criteria. First, genes had to be at least 1.28-fold overexpressed in the target cluster. Second, genes had to be expressed in more than 25% of the cells belonging to the target cluster. Third, *P*-value is less than 0.05. The False Discovery Rate (FDR) correction was used for multiple test corrections.

### Gene ontology (GO) and Kyoto encyclopedia of genes and genomes (KEGG) analyses of cell types

To annotate the function of these DEGs, GO analysis was conducted by using the GOseq software for each of the three main categories: biological process, cellular component and molecular function. Firstly, all peak related genes were mapped to GO terms in the Gene Ontology database (http://www.geneontology.org/), gene numbers were calculated for every term, significantly enriched GO terms in differentially expressed genes comparing to the genome background were defined by hypergeometric test. The calculating formula of *P*-value is:

$$P=1-\sum_{\mathrm{i}=0}^{\mathrm{m}-1}\frac{(\begin{array}{c}\mathrm{M}\\ \mathrm{i}\end{array})(\begin{array}{c}\mathrm{N}-\mathrm{M}\\ \mathrm{n}-\mathrm{i}\end{array})}{(\begin{array}{c}\mathrm{N}\\ \mathrm{n}\end{array})}$$

Here N is the number of all genes with GO annotation; n is the number of differentially expressed genes in N; M is the number of all genes that are annotated to the certain GO terms; m is the number of differentially expressed genes in M. The calculated *P*-values were corrected using FDR, taking FDR ≤ 0.05 as a threshold. GO terms meeting this condition were defined as significantly enriched GO terms in differentially expressed genes.

Genes usually interact with each other to play roles in certain biological functions. Pathway- based analysis helps to further understand genes biological functions. KEGG is the major public pathway-related database [30–32]. KEGG pathway enrichment analysis identified significantly enriched metabolic pathways or signal transduction pathways in differentially expressed genes comparing with the whole genome background results [33]. The calculating formula is the same as that in GO analysis. Here N is the number of all transcripts that with KEGG annotation, n is the number of differentially expressed genes in N, M is the number of all transcripts annotated to specific pathways, and m is number of differentially expressed genes in M. The calculated *P*-values were corrected using FDR, taking FDR ≤ 0.05 as a threshold. Pathways meeting this condition were defined as significantly enriched in differentially expressed genes.

### Statistical analysis

Statistical analysis was performed using GraphPad Prism (v8.4.0) [34]. The Shapiro–Wilk test, Wilcoxon rank-sum test, Pearson’s chi-square test, Unpaired student t test and Log-rank test were used in this study. Hazard ratio (HR) and confidence interval (CI) were calculated from Cox proportional hazards regression models. All statistical tests were two-sided, and significant differences between each two groups were indicated by *P < 0.05 and **P < 0.01.

## Results

### Liver tissue structure and cell number

**Fig. 1:**
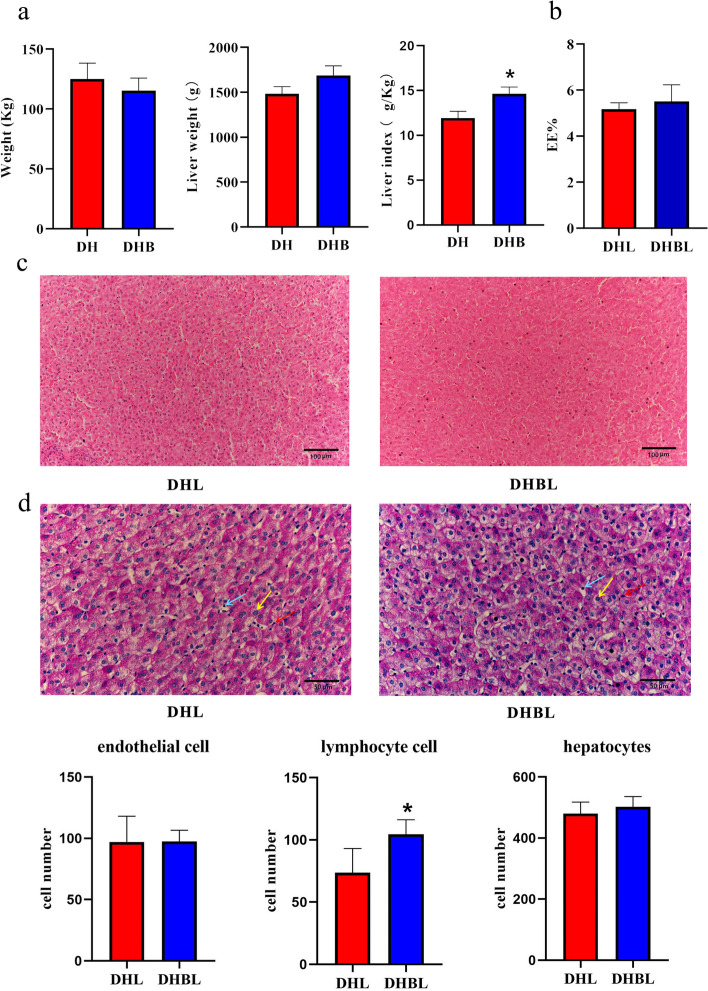
Structure features, index and the number of some cells in the DHL and DHBL. **a** The body weight, liver weight, and liver index; **b** The liver EE%; **c** liver morphology and structure with H&E. Scale bar, 100 µm; **d** Cell number in the liver tissue. Endothelial cells (red arrows), lymphocytes (blue arrows), and hepatocytes (yellow arrows). Scale bar, 50 µm

The body weight of the Dahe pigs (DH) were higher than the Dahe black pigs (DHB), while the liver weight and liver index of DH were lower than the DHB (*P* < 0.05) (Fig. 1a). Conversely, there was no significant difference in crude fat (EE) content between Dahe pig liver (DHL) and Dahe black pig liver (DHBL) (*P* > 0.05) (Fig. 1b). Liver tissues samples displayed a normal structure without obvious inflammatory cell infiltration and fibrous tissue deposition in the portal area (Fig. 1c). The number of liver tissue cells was verified using PAS, focusing on endothelial cells, lymphocytes, and hepatocytes, which were the highest numbers. Among them, there was no significant difference in the number of endothelial cells and hepatocytes of the DHL and DHBL, while the number of lymphocytes was significantly higher in the DHBL than that in the DHL (*P* < 0.05) (Fig. 1d).

### Landscape of single-nucleus transcriptome in the porcine hepatic tissue

**Fig. 2:**
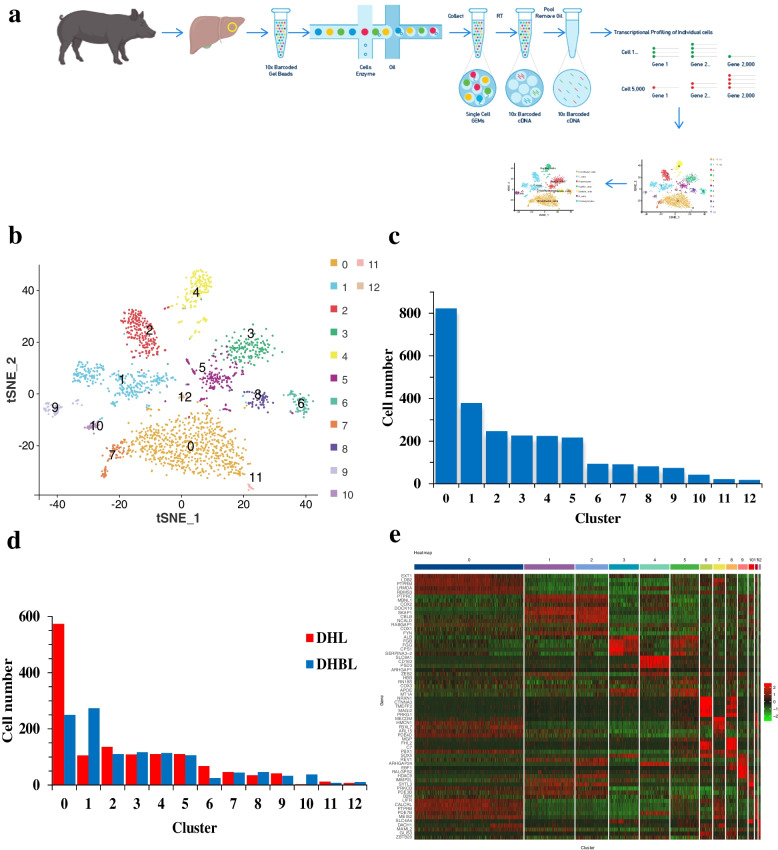
Landscape of snRNA-seq from the porcine hepatic tissue. **a** Overall strategy for snRNA-seq analyses; **b** Thirteen Clusters were obtained by the unsupervised clustering using Seurat and visualized using t-SNE; **c** Number of cells in each cluster for all samples together. **d** Number of cells in each cluster of the DHL and DHBL; **e** Heat map of the top 5 expression genes from each cluster. Each column in the figure represents a cell, and each row represents a gene. Different colors indicate the expression levels of genes in different cells. The redder, the higher the expression level; the greener, the lower the expression level

**Fig. 3:**
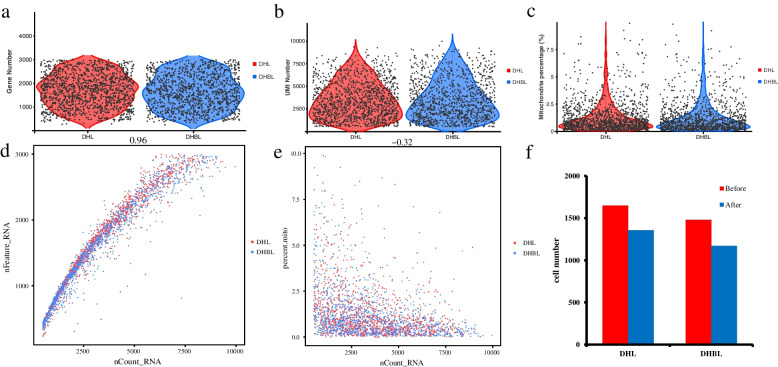
Quality control of snRNA-seq. **a** Cells with gene numbers from 240 to 3,600 were retained. The cells with fewer than 240 genes are considered low quality, and the cells with more than 3,600 genes are likely to be two or more cells in one drop; **b** Cells with 16,000 UMIs were retained. The cells with UMI numbers more than 16,000 are likely two or more cells in one drop; **c** Cells with 10% mitochondrial gene expression were retained. The percentage of mitochondrial gene expression more than 10% in a single cell indicates poor cell state, which is not conducive to subsequent analysis to reflect the real cell condition; **d** Relationship between nUMI and nGene. The dots in different colors represent cells from different samples. X axis is the number of UMI and Y axis is the number of genes percentage. The number at the top of the figure is the Pearson correlation coefficient between the number of UMI and the number of genes/mitochondria percentage; **e** Relationship between nUMI and pMito. The dots in different colors represent cells from different samples. X axis is the number of UMI and Y axis is the percentage of mitochondria. The number at the top of the figure is the Pearson correlation coefficient between the number of UMI and the percentage of mitochondria; **f** Quantity comparison before and after cell quality control

The study used snRNA-seq of liver samples to characterize cellular heterogeneity (Fig. 2a). 1,647 and 1,481 cells were captured from DHL sample and DHBL sample for library construction and paired-end sequencing. Reads from the cells with gene numbers less than 240 and over 3,600 (Fig. 3a), with more than 16,000 UMIs (Fig. 3b), and with more than 10% mitochondrial gene expression (Fig. 3c) were removed. Finally, 1,355 and 1,171 high-quality cells were obtained from the DHL and DHBL (Fig. 3d-f). 407,797,286 and 403,918,586 sequencing reads were obtained from DHL and DHBL, with 96.9% valid barcodes. 91.5% of the sequencing reads could be mapped to the DHL genome, and 92.3% of the sequencing reads could be mapped to the DHBL genome (Table S1). This study identified 23,402 genes in these 1,355 cells of the DHL, with 1,890 median genes per cell, and the median UMI count per cell was 3,767. Moreover, the study identified 23,102 genes in these 1,171 cells of DHBL, with 1,902 median genes per cell, and the median UMI count per cell was 3,934 (Table 1).

**Table 1:** Basic statistics information of snRNA-seq results

Index	DHL	DHBL
Number of Reads	407,797,286	403,918,586
Number of cells	1,355	1,171
Number of UMI	4,716,583	4,132,025
UMI/cells	3,480.87	3,528.63
Mitochondrial UMI ratio	1.28%	1.28%
Median UMI Counts per Cell	3767	3934
Number of identified genes	23,402	23,120
Median Genes per Cell	1890	1902
Sample saturation	89.00%	91.00%
Valid Barcodes	96.90%	96.90%
Q30 Bases in Barcode	95.90%	95.70%
Q30 Bases in RNA Read	90.50%	88.90%
Q30 Bases in UMI	95.00%	94.30%
Reads Mapped Confidently to Genome	91.50%	92.30%

This study classified cell types for all samples together and based on T-distributed stochastic neighbor embedding (t-SNE) dimensionality reduction and unsupervised cell clustering. Thirteen cell clusters were identified based on the expressed unique transcriptional profiles (Fig. 2b). The number of expressed genes in each cluster ranged from 9,141 to 20,115 (Table S1). The cell numbers distributed in each cluster ranged from 17 to 822 (Fig. 2c) and exhibited differences between the DHL and DHBL (Fig. 2d, Table S2). Clusters 0 and 1 were the two clusters with higher number of cells in the DHL and DHBL. Cell number of clusters 0, 2, 5, 6, 7, 9, and 11 in the DHL were higher than that in the DHBL, while Cell number of clusters 1, 3, 4, 8, 10, and 12 in the DHL were lower than that in the DHBL. Additionally, the gene expression heat map was generated for the top 5 marker genes in 13 clusters and identified the genes which were significantly enriched in each of the 13 clusters (Fig. 2e and Table S3). The results indicated certain demarcation boundaries between each cluster (Fig. 2e). Among them, cluster 1 and 2 were inferred to be a cell type preliminarily. Cluster 3 and 5 were inferred to be a cell type preliminarily.

### Identification of cell types in the porcine liver tissue

**Fig. 4:**
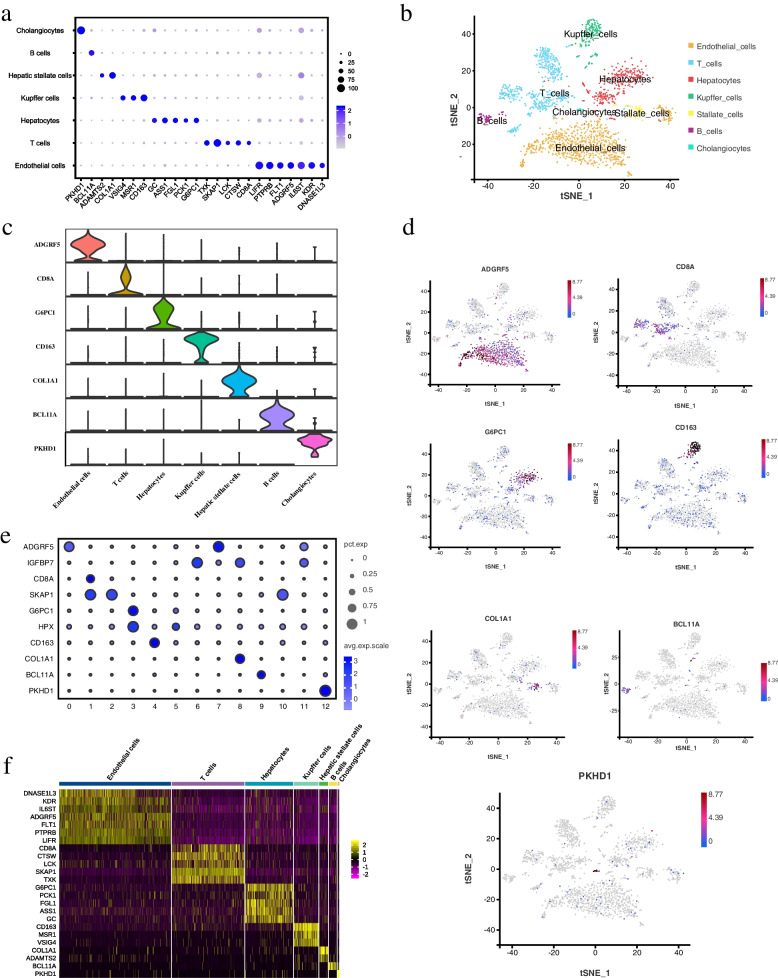
Porcine hepatic cell types analysis. **a** Circle plots indicate the expression levels of marker genes for each cell type. Normalized average UMI values for each cell type were represented by dot size and color intensity; **b** t-SNE visualization of hepatic cell types based on 2,526 single cell transcriptomes; **c** Violin plots showing the level of representative marker genes for each cell type; **d** t-SNE visualization and marker genes expression in seven cell types; **e** Circle plot of marker gene expression level in each cluster; **f** Heat map of the marker genes expression from seven cell types

Subsequent annotations were made based on the expression of cell type specific marker genes in relevant liver studies [35–40]. The thirteen cell clusters were identified as seven cell types (Fig. 4a), including endothelial cells, T cells, hepatocytes, Kupffer cells, stellate cells, B cells, and cholangiocytes (Fig. 4b). Endothelial cells were marked by adhesion G protein-coupled receptor (*ADGRF5*) and kinase insert domain receptor (*KDR*), and T cells were marked by cluster of differentiation 8 subunit alpha (*CD8A*) (Figs. 4c, d). Hepatocytes and Kupffer cells were marked by glucose-6-phosphatase catalytic subunit 1 (*G6PC1*) and cluster of differentiation 163 (*CD163*), respectively. Stellate cells and B cells were marked by collagen type I alpha 1 chain (*COL1A1*) and BCL11 transcription factor A (*BCL11A*), respectively. Cholangiocytes were marked by PKHD1 ciliary IPT domain containing fibrocystin (*PKHD1*) (Fig. 4c, d). The expression levels of each marker gene in each cell type are shown in Table 2.

**Table 2:** The expression levels of each marker gene in each cell type

Gene ID	Marker gene	endothelial cell	T cell	hepatocyte	Kupffer cell	stellate cell	B cell	cholangiocyte
ENSSSCG00000001725	*ADGRF5*	37.89	1.45	3.07	0.34	0.24	0.35	0.78
ENSSSCG00000008844	*KDR*	17.40	1.74	3.88	0.36	0.52	0.74	1.32
ENSSSCG00000008217	*CD8A*	0.35	7.00	0.60	0.10	0.15	0.45	0.00
ENSSSCG00000036449	*G6PC1*	1.14	1.16	20.28	0.59	0.76	0.15	0.86
ENSSSCG00000033146	*CD163*	1.63	3.34	3.14	39.26	0.90	0.55	1.54
ENSSSCG00000036135	*COL1A1*	1.20	0.53	1.35	0.14	12.98	0.02	0.14
ENSSSCG00000008392	*BCL11A*	0.12	0.26	0.20	2.10	0.04	7.09	0.49
ENSSSCG00000001746	*PKHD1*	0.23	0.20	0.97	0.03	0.07	0.00	44.54

**Fig. 5:**
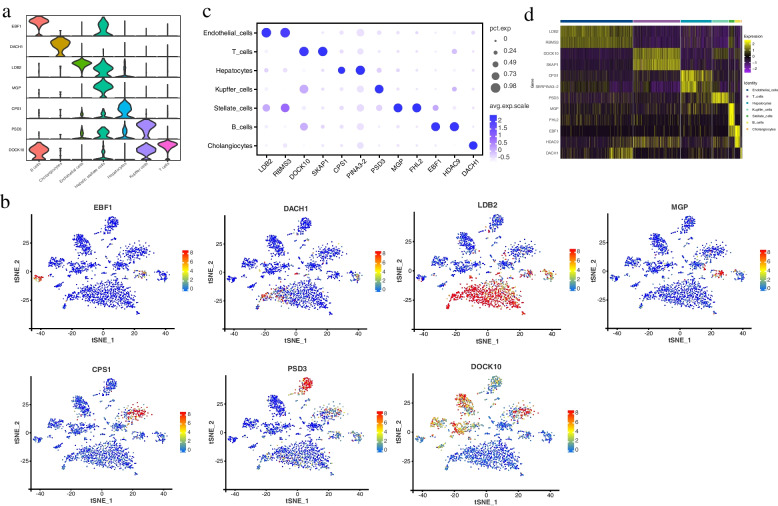
Porcine hepatic cell potential marker genes analysis. **a** Violin plots showing potential marker gene expression for each cluster; **b** t-SNE visualization and potential marker gene expression in seven cell types; **c** Circle plots illustrating subtype-specific potential marker gene expression; **d** Heatmap of potential marker expression genes from seven cell types

**Fig. 6:**
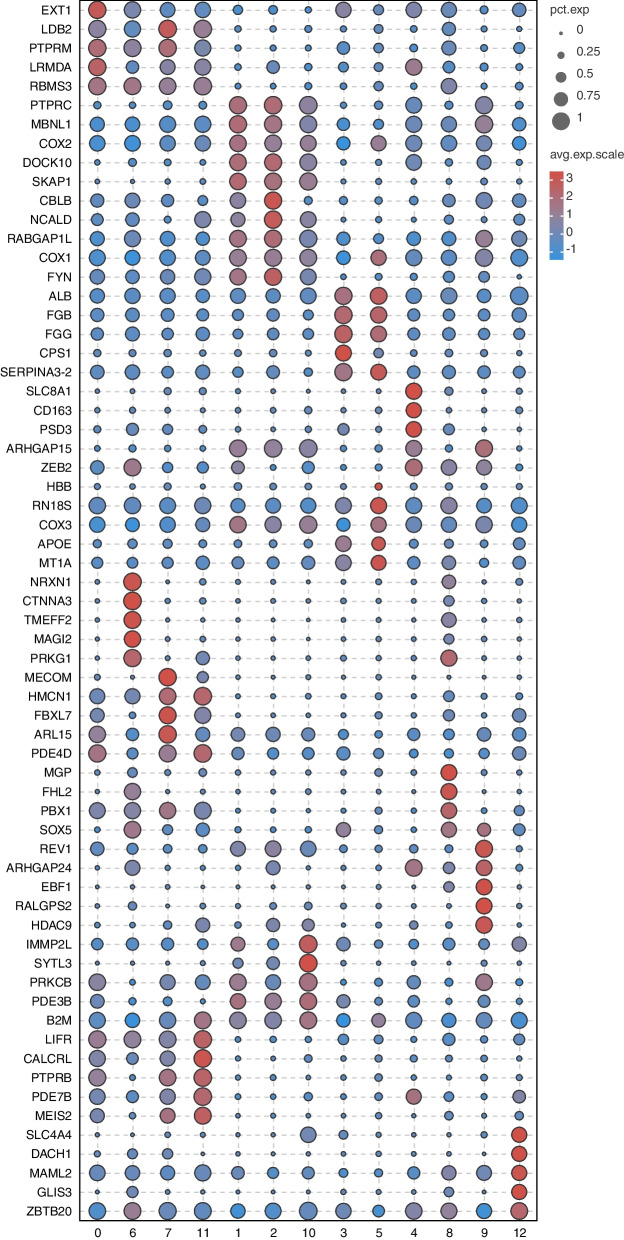
Identification of potential marker genes for seven cell types. The proportion of genes expressed in each cluster were represented by circle size. The normalized average expression level of genes in each cluster were represented by circle color

According to the circle plots, clusters 0, 6, 7, and 11 were classified as endothelial cells. Clusters 1, 2, and 10 were classified as T cells. Clusters 3 and 5 were classified as hepatocytes. Cluster 4 and 8 were classified as Kupffer cells and stellate cells, respectively. Clusters 9 and 12 were classified as B cells and cholangiocytes, respectively (Fig. 4e). A gene expression heat map of marker genes across the seven cell types was generated (Fig. 4f, Table S4). In addition, potential new marker genes for each cell type were found basing on their the highest gene expression level, such as LIM domain binding 2 (*LDB2*) and RNA binding motif single stranded interacting protein 3 (*RBMS3*) in endothelial cells, dedicator of cytokinesis 10 (*DOCK10*) and src kinase associated phosphoprotein 1 (*SKAP1*) in T cells, carbamoyl-phosphate synthase 1 (*CPS1*) and alpha-1-antichymotrypsin 2 (*SERPINA3-2*) in hepatocytes, pleckstrin and Sec7 domain containing 3 (*PSD3*) in Kupffer cells, matrix Gla protein (*MGP*) and four and a half LIM domains 2 (*FHL2*) in stellate cells, EBF1 and histone deacetylase 9 (*HDAC9*) in B cells, and dachshund family transcription factor 1 (*DACH1*) in cholangiocytes (Figs. 5, 6, Table S5).

### Number and functional enrichment of cells in the porcine liver tissue

**Fig. 7:**
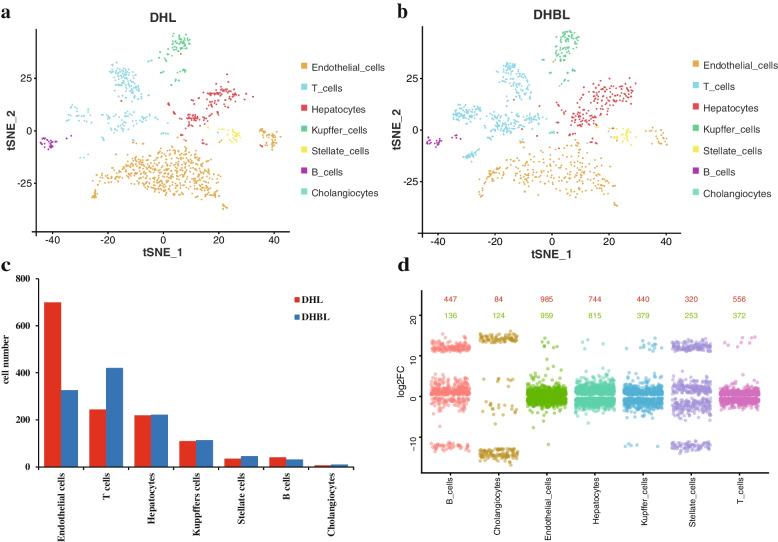
The number and function of cell in the DHL and DHBL. **a** tSNE plots showing cell numbers in distinct cell types of DHL. The cell type is color coded; **b** tSNE plots showing cell numbers in distinct cell types of DHBL. The cell type is color coded; **c** Cell number of seven cell types; **d** Cell type distribution for up-regulated (red) and down-regulated (green) genes in porcine hepatic

The study identified 699 endothelial cells, 244 T cells, 219 hepatocytes, 110 Kupffer cells, 35 stellate cells, 41 B cells, and 7 cholangiocytes in the DHL. In contrast, the study indentified 326 endothelial cells, 421 T cells, 222 hepatocytes, 114 Kupffer cells, 46 stellate cells, 32 B cells, and 10 cholangiocytes in the DHBL (Fig. 7a, b, Table S6). There were differences in the number of each cell type (Fig. 7c, Table S6). Moreover, there were DEGs between each cell type, and the number of up-regulated and down-regulated genes were shown (Fig. 7d). DEGs were obtained by comparing the gene expression between DHBL and DHL, with FC > 1.28 and *p* value < 0.05. In this study, endothelial cells, T cells and hepatocytes were the dominant cell types in the liver tissue of two pig breeds. These three cell types accounted for 85.76% and 82.74% of the DHL and DHBL, respectively. The up-regulated gene numbers in endothelial cells, T cells and hepatocytes were 985, 556, and 744 in DHBL compared to DHL, respectively. The down-regulated gene numbers in endothelial cells, T cells and hepatocytes were 959, 372, and 815 in DHBL compared to DHL, respectively (Table S7). To gain further understanding of the difference in DHL and DHBL, the study focused on the top 20 pathways.

**Fig. 8:**
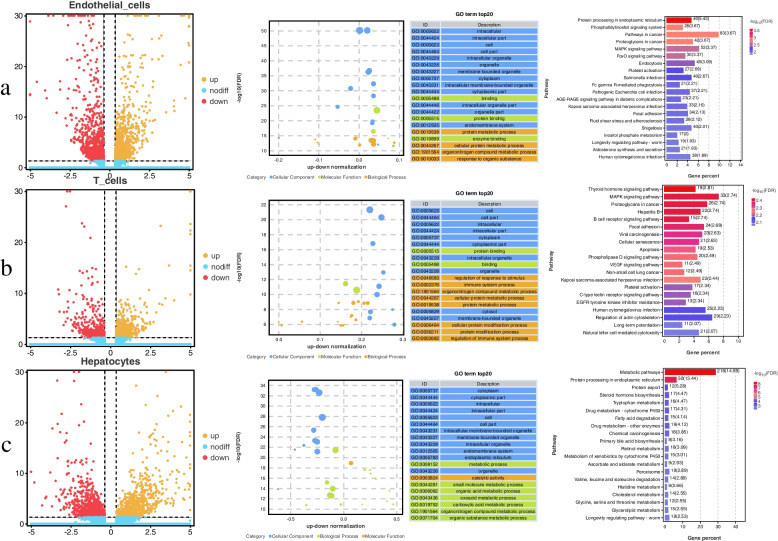
Function analysis of cells. **a** Volcano map, GO and KEGG analysis of DEGs for endothelial cells between the DHL and DHBL; **b** Volcano map, GO and KEGG analysis of DEGs for T cells between the DHL and DHBL; **c** Volcano map, GO and KEGG analysis of DEGs for hepatocytes between the DHL and DHBL. Selected top 20 with *P*-values < 0.05 are displayed

In endothelial cells, 1,944 DEGs were identified and a volcano plot of DEGs was generated (Fig. 8a). A total of 694 significantly enriched GO terms were identified, including 48 cellular component terms, 527 biological process terms, and 64 molecular function terms (Table S8). Among the top 20 terms ranked in significance, 13 terms belong to cellular components, 4 terms belong to biological process, and 3 terms belong to molecular function (Fig. 8a). A total of 20 significantly enriched KEGG pathway were identified, 10 pathways were significantly down-regulated in endothelial cell of DHBL compared to DHL, including focal adhesion, endocytosis, phosphatidylinositol signaling system, MAPK signaling pathway, FoxO signaling pathway, sphingolipid signaling pathway, phospholipase D signaling pathway, protein processing in endoplasmic reticulum, platelet activation, Fc gamma R-mediated phagocytosis, aldosterone synthesis and secretion (Table S9).

In T cells, 928 DEGs were identified and a volcano plot of DEGs was generated (Fig. 8b). A total of 498 significantly enriched GO terms were identified, including 51 cellular component terms, 399 biological process terms, and 48 molecular function terms (Table S10). Among the top 20 terms ranked in significance, 10 terms belong to cellular components, 8 terms belong to biological process, and 2 terms belong to molecular function (Fig. 8b). A total of 34 significantly enriched KEGG pathway were identified, 9 pathways were significantly up-regulated in T cells of DHBL compared to DHL. Only MAPK signaling pathway was significantly down-regulated in the T cell of the DHBL compared to the DHL (Table S11).

In hepatocytes, 1,559 DEGs were identified and a volcano plot of DEGs was generated (Fig. 8c). A total of 484 significant enriched GO terms were identified, including 77 cellular component terms, 328 biological process terms, and 79 molecular function terms (Table S12). Among the top 20 terms ranked in significance, 12 terms belong to cellular components, 7 terms belong to biological process, and 1 term belong to molecular function (Fig. 8c). A total of 30 significantly enriched KEGG pathway were identified, 27 pathways were significantly up-regulated in hepatocytes of DHBL compared to DHL, including peroxisome, ferroptosis, metabolic pathways. Protein processing in the endoplasmic reticulum and protein export were down-regulated in DHBL hepatocytes compared to DHL, respectively (Table S13).

## Discussion

The liver serves as an important metabolic organ and affects a variety of physiological functions in pigs. The molecular characteristics and functional properties of organs are determined by their constituent cell types [20]. Nevertheless, the cellular composition of pig liver remains inadequately understood. The recent advent of sensitive snRNA-seq methods has enabled the research of cell types in animal tissues [41]. To date, single-nucleus studies of human and mouse liver have been reported [14, 15, 42–44], but research of single-nucleus in porcine hepatic tissue have not been documented yet. In future research efforts, the importance of pigs is rapidly growing, because of their high homology with humans. Pigs are not only economically essential livestock, but also in anatomy, physiology, biochemistry, and drug metabolism [45–48]. Consequently, in this study, the snRNA-seq system was used to analyze cell types and functions in porcine liver tissue.

At single-cell resolution, pig liver tissue was found to contain endothelial cells, T cells, hepatocytes, Kupffer cells, stellate cells, B cells and cholangiocytes. These seven cell types are also present in human and mouse livers, suggesting conservation of major liver tissue components across species [49]. Endothelial cells form the inner wall of hepatic sinusoid, facilitating the passage of blood components and promoting cellular uptake of essential substances and secretion discharge [50]. T cells and B cells perform immune functions, while hepatocytes participate in various biological processes, from protein synthesis and lipid metabolism to detoxification [43]. Stellate cells have many functions, such as storing vitamin A, regulating hemodynamics regulation, supporting liver regeneration, and regulating immunity [51]. Kupffer cells are specialized hepatic macrophages [52], whereas cholangiocytes are bile duct-specific epithelial cells involved in bile secretion. The study revealed specific marker genes for each cell type. For example, *DOCK10* was abundantly expressed in normal T cells, and *SKAP1* acted as an immune cell adaptor, connecting the T cell receptor to LFA-1-facilitated “inside-out” signaling involved in T-cell adhesion. As a result, these genes are considered as marker genes for T cells [53, 54]. This facilitates more complex cell classification and provides new perspectives for future research on hepatic cells.

Endothelial cells, T cells and hepatocytes represent the abundant cell populations in this study. A report on mouse livers demonstrated that the highest number of hepatocytes [20]. However, in this study, endothelial cells were the most abundant, suggesting that their numbers may vary, although hepatic cell types exhibit a degree of conservation across species. Research has indicated that endothelial cells participate in angiogenesis, contraction, and vasodilation process [55]. *ADGRF5* and *KDR* genes are essential for vascular development and maintenance. In this study, *ADGRF5* and *KDR* genes were highly expressed in endothelial cells, leading us to speculate that porcine hepatic tissue may be more involved in vascular-related physiological processes. There are studies on the classification of cells in mouse liver, where endothelial cells use the *CD31* gene as a surface marker [56]. MacParland observed three endothelial cell populations, among which the most abundant endothelial cell cluster displayed enriched expression of *F8*, *PECAM1* [57]. The marker genes were used to identify liver cell types, which came from the reports in the literature and first identified in this study.

In this study, Dahe pigs were used, which are representative of typical local breeds in Yunnan, China. Dahe black pigs are a crossbreed using the Duroc × Dahe breeding scheme through five generations of selection [58]. Both varieties have the advantages of tender meat and delicious taste [59]. However, Dahe pigs grow slower and have a lower lean meat percentage, compared to Dahe black pigs [60, 61]. This study revealed differences in the cell number between the liver tissue of Dahe pig and Dahe black pig. The number of endothelial cells in liver tissue of Dahe pig was higher than that of Dahe black pig, and the opposite was true for T cells. Liver tissues of two pig breeds were analyzed by PAS staining. The numbers of endothelial cells were similar because the study observed the interior of hepatic lobules. Endothelial cells were primarily concentrated in hepatic sinusoids and interlobular connective tissue. In this area, the study observed significant differences between Dahe pigs and Dahe black pigs. The results for hepatocytes and lymphocytes were consistent with the sequencing findings, indicating that the number of hepatocytes in the liver was not significantly different, while the number of lymphocytes in Dahe black pigs was significantly higher than that in Dahe pigs.

The liver is a major metabolic organ, and its endothelial cells play a role in clearance. Sinusoidal endothelial cells, one of the most endocytotic cells in humans, display multiple scavenger receptors on their cell surface [62]. Therefore, the clearance of extracellular material depends on the health of endothelial cells and the degree of endocytic function [63]. Hepatic endothelial cells also effectively regulate the exchange of substances between hepatic sinusoidal blood flow and surrounding tissues [4]. Recent studies have demonstrated that endothelial cells and the immune environment was critical for hepatic homeostasis [64, 65]. This study provides a holistic new perspective on the processes that endothelial cell function. Endothelial cells adherently senses exogenous substances, which are then endocytosed and absorbed into the cell. Subsequently, autophagy occurs through signal transduction pathways, facilitating growth, metabolism, and immunity functions. This study revealed that the number of endothelial cells in Dahe pig liver tissue was higher than in Dahe black pig. Among the 20 pathways enriched with differentially expressed genes in hepatic endothelial cells of Dahe pig liver and Dahe black pig liver, 3 pathways were up-regulated, and 10 pathways were down-regulated of Dahe black pig liver tissue compared to Dahe pig. This observation may indicate a positive correlation between cell number and functional diversity. This study indicates that changes in the number of hepatic cells were observed after pig hybridization.

The animal liver, an organ with multiple immune functions, serves as a sentinel for the human immune system [66]. In recent years, the crosstalk between the liver and the immune system has been uncovered through the study of hepatic snRNA-seq [67, 68]. T cells play a central role in adaptive immunity, and their activation involves spatially and temporally coordinated signaling processes across multiple time and length scales [69]. This study identified T cells in the livers of two pig breeds, with T cells constituting the second largest cell population. Notably, the number of T cells in Dahe black pig liver tissue was higher than in Dahe pig the immune and endocrine functions of the liver of Dahe black pigs are potentially stronger. In the future, we need to do more research to prove this conclusion.

Hepatocytes are the major parenchymal cells of the liver. Hepatocytes play critical roles in liver homeostasis and disease development [70]. These cells are responsible for the majority of hepatic metabolic, biosynthetic, biodegradable, and secretory functions [71]. And hepatocytes are involved in biological processes ranging from protein synthesis and lipid metabolism to exogenous and endogenous detoxification [43]. Many functions of hepatocytes require close cooperation between cell adhesion molecules, cell junctions, cytoskeleton, extracellular matrix, and intracellular trafficking machinery [72]. The study revealed only small differences in the number of hepatocytes in the liver tissue of Dahe pig and Dahe black pig. Among the 30 pathways enriched with significantly different genes in hepatocytes of Dahe pig liver and Dahe black pig liver, 27 pathways were up-regulated, and 2 pathways were down-regulated of Dahe black pig liver tissue compared to Dahe pig. These results indicated that hepatocytes of Dahe black pigs exhibited more abundant metabolic and biosynthetic functions, especially in protein metabolism, lipid metabolism, amino acid metabolism, and carbohydrate metabolism. This is supported by a recent report demonstrating specificity in liver lipid metabolism between different pig breeds [73]. The findings highlight breed differences in liver tissue between Dahe pigs (a Chinese indigenous breed) and Dahe black pigs (a crossbreed using the Duroc × Dahe breeding scheme through five generations of selection). Variations in the number of cell types were detected between the two pig breeds. Considering the functional enrichment of differentially expressed genes, it can be inferred that these cell types may exhibit mutual compensatory effects.

A limitation of this study is that the small number of liver samples sequenced. So, interpretations of some functions of the liver may not generalize. In essence, leveraging single-nucleus resolution and cost-effective UMI-based approaches to enhance throughput, the study findings also furnish foundational data for future investigations in this field. In the future, functional validation of target genes will be carried out, with the initial goal of conducting validation on cells.

## Conclusions

In summary, this study establishes a comprehensive single-nucleus atlas landscape and identifies potential new marker genes for each cell type. The number, gene expression level and functional characteristics of each cell type in pig liver tissue varied between breeds. These high-quality snRNA-seq data serve as a valuable resource for future studies on porcine hepatic function and may provide informative support for human hepatic health and immunity.

### Supplementary Information

**Additional file 1. Table S1. **Gene number in each cluster.

**Additional file 2. Table S2. **Cell number in each cluster.

**Additional file 3. Table S3. **The top five expression genes from each cluster.

**Additional file 4. Table S4. **The marker genes of each cell type.

**Additional file 5. Table S5. **The new marker genes of each cell type.

**Additional file 6. Table S6. **Cell number in each cell type.

**Additional file 7. Table S7. **Number of differential genes per cell type.

**Additional file 8. Table S8. **Endothelial cells differential gene GO enrichment.

**Additional file 9. Table S9. **Endothelial cells differential gene KEGG significant enrichment（DHL-VS-DHBL).

**Additional file 10. Table S10. **T cells differential gene GO enrichment.

**Additional file 11. Table S11. **T cells differential gene KEGG significant enrichment（DHL-VS-DHBL).

**Additional file 12. Table S12. **Hepatocytes differential gene GO enrichment.

**Additional file 13. Table S13. **Hepatocytes differential gene KEGG significant enrichment（DHL-VS-DHBL).

## Data Availability

Data is contained within the article or supplementary material. Additional data that support the findings of this study are available from the corresponding author upon reasonable request. The accession number for the liver single nucleus RNA sequencing data reported in this paper is PRJNA956984.

## Abbreviations

*ADGRF5*: Adhesion G protein-coupled receptor
*BCL11A*: BCL11 transcription factor A
*CD8A*: Cluster of Differentiation 8 subunit alpha
*CD163*: Cluster of Differentiation 163
*CPS1*: Carbamoyl-phosphate synthase 1
*COL1A1*: Collagen type I alpha 1 chain
DH: Dahe pig
DHB: Dahe black pig
DHL: Dahe pig liver
DHBL: Dahe black pig liver
DEGs: Differentially expressed genes
*DOCK10*: Dedicator of cytokinesis 10
*DACH1*: Dachshund family transcription factor 1
*EBF1*: EBF transcription factor 1
FDR: False Discovery Rate
*FHL2*: Four and a half LIM domains 2
GO: Gene ontology
GEMs: Gel Bead-In-EMlusion
*G6PC1*: Glucose-6-phosphatase catalytic subunit 1
*HDAC9*: Histone deacetylase 9
KEGG: Kyoto Encyclopedia of Genes and Genomes
*KDR*: Kinase insert domain receptor
*LDB2*: LIM domain binding 2
*MGP*: Matrix Gla protein
PAS: Periodic acid-Schiff staining
PCA: Principal component analysis
*PSD3*: Pleckstrin and Sec7 domain containing 3
*PKHD1*: PKHD1 ciliary IPT domain containing fibrocystin
*RBMS3*: RNA binding motif single stranded interacting protein 3
*SKAP1*: Src kinase associated phosphoprotein 1
snRNA-seq: Single-nucleus RNA sequencing
SNN: Shared-nearest neighbour
*SERPINA3-2*: Alpha-1-antichymotrypsin 2
tSNE: T-distributed stochastic neighbor embedding
UMI: Unique Molecular Identifier
